# Status of Human Papillomavirus Infection in the Ethnic Population in Yunnan Province, China

**DOI:** 10.1155/2015/314815

**Published:** 2015-12-27

**Authors:** Zulqarnain Baloch, Lei Yue, Tao Yuan, Yue Feng, Wenlin Tai, Yanqing Liu, Binghui Wang, Xiao Li, Li Liu, A-Mei Zhang, Xiaomei Wu, Xueshan Xia

**Affiliations:** ^1^Faculty of Life Science and Technology, Kunming University of Science and Technology, Kunming 650500, China; ^2^The Research Centre for Molecular Medicine in Yunnan Province, Kunming 650500, China; ^3^The First Hospital of Yunnan Province, Kunming 650034, China; ^4^The Second Affiliated Hospital of Kunming Medical University, Kunming 650101, China

## Abstract

HPV genotypes have distinct distributions among various ethnic populations worldwide. In December 2013, 237 and 159 cervical samples were collected from Hani and Han ethnic women, respectively, in Mojiang, a rural county in southern Yunnan. The overall HPV infection rate (21.1%) among the Hani women was significantly higher than that among the Han women (12.6%). The high-risk (HR) and low-risk (LR) HPV and single- and multiple-genotype infection rates among the Hani women were 11.0%, 4.6%, 15.6%, and 5.5%, respectively. HPV-16 (3.8%) was the most prevalent genotype among the Hani women, followed by HPV-52 (1.7%), HPV-31 (0.8%), and HPV-33 (0.8%). Comparatively, the Han women had lower infection rates of high-risk (8.2%), low-risk (1.2%), single-genotype (9.4%), and multiple-genotype HPV infections (3.1%). HPV-16 (3.1%) was also the predominant genotype among the Han women, followed by HPV-52 (1.3%), HPV-33 (0.6%), HPV-44 (0.6%), and HPV-54 (0.6%). The area background, number of children, and past history of STIs were recognized as potential risk factors for HPV infection. Rural background, age, education level, number of children, and illness history were significantly associated with HPV infection among the Hani women. These findings highlight the urgent need for HPV prevention and control strategies in Yunnan, particularly for the Hani ethnic women.

## 1. Background

Human papillomavirus (HPV) is one of the most common sexually transmitted pathogens of the genital system and plays a vital role in the development of cervical cancer [1, 2]. It is well recognized that 75–80% of sexually active women are infected with HPV at some point during their life [3, 4]. Worldwide, cervical cancer caused by HPV is the second most common cancer among the female population; in particular, it is the leading female cancer in developing countries around the world [2, 5, 6]. Papillomaviruses are nonenveloped, circular, double-stranded DNA viruses with a genome length of 8 kb. HPV genotypes are classified as either high risk or low risk according to their carcinogenicity. High-risk HPV is responsible for malignant lesions and includes more than one dozen of genotypes [7, 8], while low-risk HPV may cause benign anogenital warts, skin warts, and respiratory tract infections [8, 9].

Epidemiologically, the HPV prevalence rates and genotype distributions vary among different geographical regions. The highest HPV prevalence in women without cervical abnormalities has been reported in Africa (24%), followed by Eastern Europe (21%), Latin America (16%), and Southeast Asia (14%) [10]. The HPV prevalence rates and genotype distributions also differ among the female populations of different ethnic groups in the world particularly in China; recently a study was reported by our group in which HPV prevalence was significantly high among Tibetan women (27.4%) compared to Han (17.2%) and Naxi (11.9%) women [11]. Generally, HPV-16 is the predominant high-risk genotype worldwide, while the frequencies of other genotypes vary from region to region [12]. A study conducted in Northwestern Yunnan identified a significant difference of HPV-16 and HPV-33 prevalence among Tibetan (13.0%, 6.1%) subjects compared with those of Han (3.8%, 5.1%) and Naxi (5.0%, 1.0%) group.

Cervical cancer cases are increasing in China, and due to distinct topographical variations, HPV prevalence varies among different regions of China [13]. The high-risk HPV (HR-HPV) infection rate has been estimated to range from 15.0% to 20.8%, and the mortality rate due to cervical cancer increases by 4.1% each year [14]. Pooled analysis of 17 different populations has reported a 17.7% high-risk human papillomavirus (HR-HPV prevalence corresponding to genotypes 16, 18, 52, 58, and 59) [15, 16]. Although two prophylactic HPV vaccines have been approved in >140 countries, they were not approved by the Chinese government due to an absence of Chinese patient clinical data [17]. In 2008, two WHO-recommended vaccine companies began phase III Chinese clinical trials, and it is expected that they will obtain approval very soon [18]. Considering the high variations in the HPV prevalence rates and genotype distributions in China, investigation of these parameters in various Chinese populations would be very useful for designing HPV control strategies.

Hani group is a Chinese certified ethnic minority group that mainly inhabits southeast China [19]. Additionally, Hani group is a certified ethnic minority group in some Southeast Asian countries, where they are referred to as Akha or Houni [20]. According to the national census figures (http://www.hxen.com/word/xinwen/2011-04-28/142164.html), the size of the Hani ethnic population living in Yunnan is 1.63 M, making it the third largest local population. The majority of individuals in the Hani population are illiterate, have a high fertility rate, mainly work in agricultural farming, and live in remote mountainous areas with poor sanitary conditions [21]. Han group represents the ethnic majority, comprising 92% of the total population of China, and they are equally distributed throughout China. They are well educated, mostly belonging to the ruling class, and they reside in urban areas. The purpose of the current study was to comparatively investigate the HPV prevalence rates and genotype distributions and the risk factors for HPV infection among women of the Hani ethnic minority and the Han majority in southern Yunnan, China.

## 2. Materials and Methods

### 2.1. Participant Recruiting

In June and July of 2014, this cross-sectional study was collaboratively conducted at Mojiang by the Department of Gynaecology, the First People's Hospital of Yunnan Province, and Kunming University of Science and Technology. Interested women were requested to visit a local hospital in Mojiang County, Yunnan, and written consent was obtained. A standardized questionnaire was used to collect information about ethnic background, age, education, marital status, illness history, sexual activity, and profession. After completion of the questionnaire, a qualified gynaecologist performed pelvic examination and sample collection. Women who met the following criteria were recruited into the current study: those who (a) were not a gynaecological outpatient with symptoms related to genital tract disease (commonly cervicitis and/or vulvar discomfort); (b) were not currently pregnant; and (c) had not undergone a total uterus or cervical resection. Finally, a total of 396 participants from general population, including 237 Hani and 159 Han women, were considered qualified and were recruited for this study. The protocol was followed in accordance with the Declaration of Helsinki and was approved by the Ethics Committee of Kunming University of Science and Technology and the Center for Disease Control and Prevention (CDC) in Yunnan Province, China.

### 2.2. Cytological Analysis

A qualified gynaecologist collected exfoliated cervical cells from each participant using a cytobrush (Hybribio). Each sample of exfoliated cervical cells was inserted into a vial with preservation solution (Hybribio, Chaozhou, China) and vigorously swirled 10 times. The vial contents were sent to Yunnan First People's Hospital for cytological analysis. All cytological slides were individually prepared by two qualified technicians. All of the cytology specimens were classified according to the Bethesda classification system [22]. All smears were diagnosed without abnormalities.

### 2.3. DNA Extraction

Cervical samples were collected with a cervix brush, and cells were placed into a vial containing preserving media. The samples were transported at −20°C to a central laboratory. A QIAamp DNA Mini Kit (Qiagen, Valencia, CA, USA) was used to extract DNA from the cervical samples according to the manufacturer's instructions.

### 2.4. HPV Detection and Genotyping

PCR amplification of the housekeeping *β*-globin gene was performed to evaluate DNA quality [23]. Samples that showed *β*-globin positivity were further analyzed by PCR amplification of HPV DNA. HPV-positive samples were confirmed by PCR with universal L1 primers using the MY09/11 and GP5/6 systems [24]. DNA extracts from HeLa and CaSki cell lines were used as positive controls, and mixtures without sample DNA were used as negative controls.

HPV genotypes were determined using an HPV GenoArray Test Kit (Hybribio, Chaozhou, China) according to the manufacturer's instructions. The GenoArray test is an L1 consensus primer-based PCR assay that is capable of amplifying 21 HPV genotypes, including 15 HR-HPV genotypes (16, 18, 31, 33, 35, 39, 45, 51, 52, 53, 56, 58, 59, 66, and 68) and 6 low-risk HPV genotypes (LR-HPV) (6, 11, 42, 43, 44, and CP8304) [25]. The assay was conducted according to the manufacturer's recommendations. Samples that were identified as positive but were not typed by the GenoArray test were submitted for direct sequencing by a commercial company (Invitrogen, Beijing, CN). All the samples that were identified as positive through PCR were genotype with two methods. First we run GenoArray test and secondly we performed direct sequencing. For the DNA sequencing, new PCR was run with reaction mixture 50 *μ*L containing 4 *μ*L of the DNA extract, 2 *μ*L of 10 *μ*molar MY09 primer, 2 *μ*L of 10 *μ*molar MY11 primer and 25 *μ*L of the provided master mixture, and 17 *μ*L dH_2_O. The consensus primer products were separated by electrophoresis on a 2% agarose gel and purified with Tiangel PCR purification kit. The DNA was then directly sequenced using the ABI PRISM Big Dye Terminator Cycle Sequencing Ready Reaction (Invitrogen, Beijing, CN) on an ABI 310 DNA analyzer. The nucleotide sequences were aligned and compared with those of known HPV genotypes available through Genbank by using the BLAST 2.0 software server (http://blast.ncbi.nlm.nih.gov/Blast.cgi). The sample was identified as a particular HPV genotype if the sequence was 95% homologous with the reference standard.

### 2.5. Statistical Analysis

The HPV prevalence rates, including the total rates, and the rates for single- and multiple-genotype infections, were compared using the Chi-square test. Corresponding 95% confidence intervals (CIs) were estimated by binomial distribution analysis. The effects of age-related variables on HPV infection in women were assessed using the logistic regression model, with the grouping of age in 10-year intervals (<35, 36–45, and <46), and odds ratios and their 95% CIs were calculated. Possible risk factors for HPV infection acquisition were evaluated by univariate analysis and multivariate logistic regression analyses, and odds ratios and their 95% CIs were calculated. A *P* value of 0.05 was considered statistically significant. The results of all manual statistical analyses were confirmed with SPSS software package (version 20.0).

## 3. Results

### 3.1. Overall HPV Prevalence

Among the 396 recruited women, 70 were found to be HPV DNA positive (17.7%, 70/396). Of them, 52 (13.1%) were positive for an HPV single-genotype infection, while 18 (4.5%) had a multiple-genotype infection. A total of 23 genotypes were identified, with the most frequently detected HR-HPV genotypes being HPV-16 (14/396, 3.5%), HPV-52 (6/396, 1.5%), HPV-33 (3/396, 0.8%), HPV-58 (2/396, 0.5%), and HPV-18 (2/396, 0.5%). Comparatively, the most common low-risk HPV genotypes were HPV-54 (3/398, 0.8%), HPV-11 (3/396, 0.8%), HPV-55 (2/396, 0.5%), and HPV-42 (2/396, 0.5%). The prevalence of HR-HPV (9.8%, 39/396) was significantly higher than that of low-risk human papillomavirus (LR-HPV) (3.3%, 13/396), with a *P* value of 0.048 (Table 1).

Coinfection of multiple HPV genotypes did not occur frequently. In total, 4.5% (18/396) of the patients had a multiple-genotype HPV infection. Of them, 3.5% (14/396) had a double infection (HPV-16/52, HPV-52/6, and HPV-54/55), 0.8% (3/396) had a triple infection (HPV-33/35/58, HPV-16/31/52, and HPV-16/58/44), and only 0.2% were found to be infected with four genotypes (HPV-16/33/11/40) (Table 1). Among these patients with multiple infections, 10/396 (2.5%) presented with a multiple HR-HPV infection, only one exhibited coinfection with multiple LR-HPV genotypes, and 7/396 (1.8%) were found to be coinfected with both HR-HPV and LR-HPV genotypes. HPV-16, HPV-52, and HPV-58 appeared to be responsible for most of the multiple-genotype infections (3.3%, 13/396).

### 3.2. Higher HPV Prevalence and Genotype Distribution among the Hani Women

Among the 237 recruited Hani women, 50 (50/237, 21.1%) were found to be HPV DNA positive. In contrast, HPV DNA was detected in only 12.6% (20/159) of the Han women. The HPV prevalence observed among the Hani women (21.1%) was significantly higher than that among the Han women (12.6%), with a *P* value of 0.032. As the primary infection form, the HPV single-genotype infection rate among the Hani women (15.6%, 38/237) was significantly higher (*P* = 0.016) than that among the Han women (9.4%, 14/159). The multiple infection rate among the Hani women (13/237, 5.5%) was also higher than that among the Han women (5/159, 3.1%). Furthermore, the average percentage of samples containing at least one HR-HPV genotype was higher among the Hani women (11.0%) than among the Han women (8.2%), although this difference was not significant (*P* = 0.35). The LR-HPV prevalence among the Hani women was 4.6% higher than that among the Han women (1.3%; *P* = 0.084).

**Figure 1:**
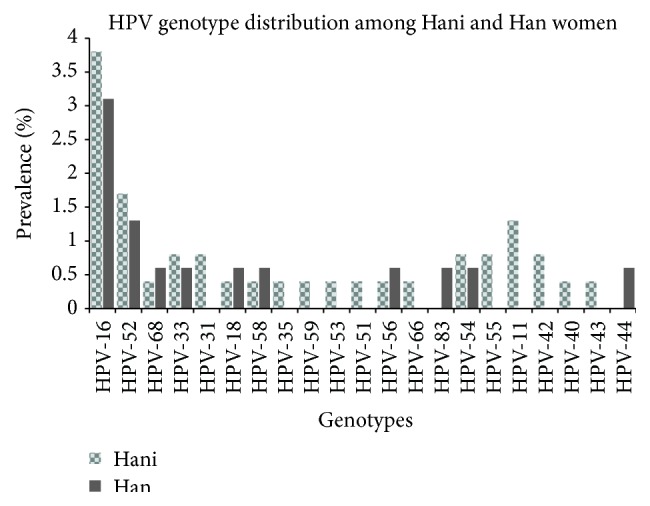
Human papillomavirus genotype-specific distribution (Yunnan, China, 2015).

A total of 23 HPV genotypes were identified in this study (Figure 1). Of them, 8 (HPV-16, HPV-52, HPV-33, HPV-18, HPV-54, HPV-56, HPV-58, and HPV-68) were detected in both ethnic groups; these genotypes were responsible for 8.9% (21/237) of the infections among the Hani women and 8.2% (13/159) of those among the Han women. Two genotypes, HPV-39 and HPV-6, were only identified in multiple infections among the Hani women. Interestingly, some of the genotypes that were not found to be causes of single-genotype infections in either ethnic group were observed in multiple infections. For example, HPV-44 infection alone was not found in the Hani group, but this genotype was detected in multiple infections in that group, while HPV-31, HPV-43, and HPV-66 were observed in multiple-genotype infections among the Han women.

### 3.3. The Effect of Age on HPV Prevalence and Other Risk Factors

**Figure 2:**
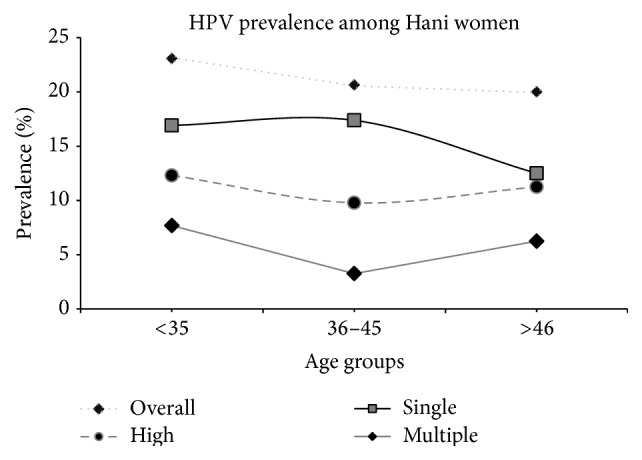
Age-specific prevalence of human papillomavirus DNA and corresponding 95% confidence interval (Hani, Yunnan, China, 2015).

The mean age of the 396 recruited women was 41.58, with a range of 18 to 74 years (SD = 7.5 years, 95% CI, 40.92–42.41) and median of 41 years. There was no significant age difference between the Hani (41.53 years (median = 41), SD = 8.79, 95% CI, 40.41–42.66) and Han ethnic groups (41.86 years (median = 41), SD = 4.99, 95% CI, 41.08–42.64) (*P* = 0.67). Correlation analysis revealed that HPV prevalence was more complex in the younger and older participants compared with the middle age group. The highest overall HPV prevalence was observed in the <35-year age group, and it declined thereafter with increasing age. A less pronounced second peak in prevalence was observed for the Han women in the older age group (>46 years). However, high-risk and multiple-genotype infections among the Hani women also exhibited two peaks in prevalence, with the first peak of 12.3% and 7.7%, respectively, occurring at <36 years and the second peak of 11.2% and 6.2% occurring at >46 years. The prevalence of single-genotype infections among the Hani women peaked in the middle age group and declined thereafter as age increased (Figure 2). However, high-risk infections among the Han women were the most prevalent in the <35 years age group, and the prevalence declined thereafter as age increased (Table 2).

Multivariate logistic regression analysis was applied to determine the roles of various possible risk factors in the acquisition of HPV infection. Marital status and extra sexual partners were not found to significantly contribute to the development of HPV infection for either group of participants. Notably, we cannot draw any conclusions about the role of sexual activity in HPV infection because 98.5% of the participants were married and had a single sexual partner. However, the Hani women and the women who had a rural background and multiple children and a history of sexually transmitted infections (STIs) had a significantly higher risk of being infected with HPV (Table 3).

## 4. Discussion

Cervical malignancy is the 8th most common cancer among Chinese females [14]. It is well known that mass immunization with HPV vaccines has the potential to minimize the incidence of cervical cancer. Two HPV prophylactic vaccines have been approved in >140 countries; however, they were not approved by the Chinese government due to an absence of Chinese patient clinical data [17]. A vaccine undergoing phase III trials in China has been formulated based on data obtained from developed areas. Yunnan Province has a unique geographical location, highly complex topography, and large variations in elevation. Particularly, twenty-six state-certified ethnic populations are scattered across the remote and hilly region of Yunnan. The health care facilities in this region are inadequate, and there is a lack of cancer registries and gynaecological screening and HPV testing programs [26]. Knowledge of HPV prevalence rates and genotype distributions among ethnic populations is important for the design of preventative strategies at the local or national level.

In this study, the overall HPV infection rate of 17.6% was higher than the documented rates in neighbouring countries (6.2% in Southeast Asia, 6.6% in south central Asia, and 8.0% in other Asian countries) [27, 28]. However, reports from Northwestern Yunnan and some regions of mainland China have stated that the overall prevalence is 18.4% in Northwestern Yunnan [11], 13.3% in Zhejiang province [29], and 14.8% in Shanxi province [30], in agreement with our reported HPV prevalence. Hani group is a Chinese ethnic minority group that primarily inhabits Yunnan and Southeast Asian countries. Anthropologically, individuals in this ethnic group are descendants of the ancient Qiang people of northwest China or Sichuan [17] who migrated to and populated the Ailao and Mengle mountains from the 4th to the 8th century BC [19]. The reported HPV prevalence among Han women throughout China ranges from 6.7% in Beijing to 29.6% in Shanxi [30, 31]. The rate (12.6%) for the Han women in this study was also within this range. However, the HPV prevalence among the Hani women (21.1%) was significantly (*P* < 0.05) higher than that among the Han women (12.6%). The prevalence of HPV among Hani is lower than results among Tibetan women (27.4%) from Northwestern Yunnan and is higher compared with Naxi (11.9%) and Han (17.2%) cases [11]. We suspect that genetic and social backgrounds and cultural traits might play vital roles in HPV prevalence because the Han and Hani women live under the same sanitary conditions.

HR-HPV prevalence has been examined in previous investigations due to its vital role in the development of cervical cancer [32]. This prevalence has been documented to vary in different geographic locations; for example, it is 8.1% in Europe, 11.3% in North America, 22.1% in Africa, and 8.0% in Asia [28]. Here, we found that its prevalence was much higher among the Hani women (11.0%) than the Han women (8.2%). The HR-HPV prevalence in the Han women was in line with the previously reported infection rate for Qujing City, Yunnan (8.3%), and the Autonomous Tibet (8.0%) prefecture [26, 33]. However, the HR-HPV prevalence in the Hani women was higher compared with other ethnic minority groups in China, such as the Uyghur (7.3%) [34] and Maonan (7.6%) group, [35] but lower than Tibetan (26.2%) and Han (16.6%) groups in Northwestern Yunnan. However, the reported HR-HPV prevalence rates in Naxi group (11.4%) in Northwestern Yunnan [11], Zhejiang (10.2%), and Shanxi (12.2%) are in line with our observations [29, 30]. The LR-HPV prevalence rates have been reported to be 3.8% and 4.4% in Shanxi and Taiwan, respectively [30, 36], in accordance with our identified LR-HPV prevalence of 4.6% among the Hani women. Comparatively, the calculated LR-HPV prevalence rate among the Han women is similar to those in areas with lower LR-HPV prevalence rates in China, such as Shandong and Tibet [26, 37]. Multiple infections with different HPV genotypes can raise the risk of cervical cancer compared with single-genotype infections [32]. It has been reported that the multiple-genotype infection rate ranges from 4 to 15.7% [7]. In the current study, the HPV multiple-genotype infection rate among the Hani women was 5.5%, and this rate is higher in other regions of Asia and Europe [38, 39]. However, the multiple infection prevalence rate among the Han women was 3.1%, which is in agreement with the reported rates in other regions of China (3.2%) [38].

An understanding of HPV genotype distributions in different areas or populations is crucial for HPV prevention and vaccine development. In this study, HPV-16 (3.5%) was detected as the most frequent genotype among the women in both ethnic groups, but it was more common among the Hani women (3.8%) than the Han women (3.1%). However, coinfection with HPV-18, which is considered a highly carcinogenic genotype, and HPV-16 was not frequently detected in this study [37, 40]. HPV-52 was identified as the second most prevalent genotype, in agreement with previous reports from Asian countries, particularly China [41, 42]. The distributions of the remaining HPV genotypes exhibited some differences between the two ethnic groups. For example, HPV-31 and HPV-33 were the third most common infections among the Hani women; however, these genotypes were not identified in the Han group. Instead, HPV-44 and HPV-83 were more common among the Han women. These findings indicate that the HPV genotype distribution varies even within the same city or population [32].

**Figure 3:**
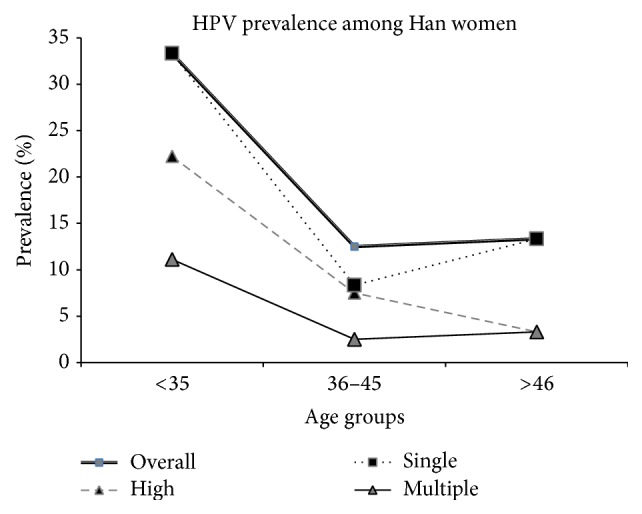
Age-specific prevalence of human papillomavirus DNA and corresponding 95% confidence interval (Han, Yunnan, China, 2015).

Age is another important factor affecting HPV prevalence due to the various physiological developmental stages. A cross-sectional study of women from Hong Kong has shown that an initial infection peak occurs at young ages, with a second peak occurring in women over 60 years of age, while a single peak in HPV prevalence has been detected in 20- to 29-year-old Guangzhou women. Thereafter, the prevalence has been shown to decrease with increase in age [43]. Another epidemiological study has indicated that the prevalence of high-risk HPV rises to an initial peak at a young age, with an infection rate of 13.3–15%, and a second peak emerges at a later age, with an infection rate of 17–24% [14]. In this study, “U-” shaped (Figure 3) age-specific prevalence rates were identified for the overall HPV prevalence and multiple-genotype infection prevalence among the women from both ethnic groups. The HPV infection rate increased again to form a second peak in the older age group (>46 years). This pattern has been reported in previous investigations [33, 42]. The frequently occurring HPV infections among young females may be caused by the initiation of sexual activity, while the second peak may occur due to decreased clearance, the persistence of HPV infection, or changes in sexual behaviour [43, 44]. In contrast, a low prevalence rate was observed among the middle age group (36–45 years), which may be due to the development of the immune systems of infected women in this age range, which protects them from infections.

Women residing in a rural area were found to be at high risk of HPV infection. Most of the Hani women were from rural areas, where they lived in concentrated villages. The sanitary conditions in their houses were found to be the worst. Further, medical hospitals in these areas are lacking, and if inhabitants become sick, they prefer to be treated at home with their traditional medicine. A possible interpretation of these findings is that women living in villages without health care facilities may be at high risk of HPV infection and the development of cervical cancer. It is therefore crucial to identify these populations and to stress the importance of cervical screening. Various reproductive characteristics are linked to the acquisition of HPV infection. In this study, Hani women with a past history of sexually transmitted diseases and those with multiple children were at significantly higher risk of HPV infection. This finding suggests that sexually transmitted diseases may also be a risk factor for HPV infection in other populations [45, 46]. Thus, the results of the present study suggest that women who are infected with STIs are at a significantly increased risk of HPV infection and should be intermittently screened because they are considered at risk of the development of cervical cancer. Both of the ethnic groups included in this study resided together in the same areas; however, their socioeconomic conditions, including their lifestyles and living standards, were quite different. The Han population is a well-educated and developed ethnic group compared with the Hani minority. The higher HPV infection prevalence among the Hani women needs further confirmation with consideration of their lifestyles and traditions, which may influence HPV prevalence.

We have identified different HPV prevalence rates and genotype distributions among the Hani and Han women and have described the roles of different potential risk factors in the acquisition of HPV infection. Further, we have determined the age-specific HPV infection rates among these two groups; however, there are still some limitations to this study. For example, the number of participants is low, particularly the Han women (<35 years, >46 years). Thus, the results generated from the two groups are unreliable, which limits the generalizability of the findings. In view of the findings of previous studies indicating that the older age group is at high risk of acquiring HPV infection due to their compromised immune systems, it is crucial to perform further investigations to clarify our results.

## 5. Conclusions

To our knowledge, this is the first Hani ethnic population-based study conducted in Yunnan, China. Significantly higher prevalence rates of both general HPV and single HPV genotype infection were discovered among the Hani women compared with the Han women. A young age was found to be a risk factor for acquiring HPV infection. In addition, residing in a rural area, a past history of STIs, and a history of multiple pregnancies were found to be associated with acquisition of HPV infection. These findings contribute to the understanding of the epidemiological characteristics of HPV among women in different ethnic groups and will facilitate the design of effective strategies for the prevention and control of HPV.

## Figures and Tables

**Table 1:** The prevalence of HPV and genotypes distribution among Hani and Han ethnic women.

Variables	Total (*n* ^1^ = 396)		Hani (*n* ^1^ = 237)		Han (*n* ^1^ = 159)		*P* value^2^
	Positive	P% (95% CI)^3^	Positive	P% (95% CI)^3^	Positive	P% (95% CI)^3^	
Age	41.67 ± 7.62 (40.92–42.42)		41.58 ± 8.99 (40.43–42.73)		41.81 ± 4.91 (41.04–42.58)		0.76
Prevalence	70	17.68 (13.93–21.44)	50	21.1 (15.91–26.29)	20	12.6 (7.45–17.75)	**0.032**
Single infection	52	13.13 (9.8–16.46)	38	15.6 (11.4–20.22)	14	9.4 (5.06–14.14)	**0.016**
High risk	38	9.6 (6.7–12.5)	26	11 (7.01–14.98)	13	8.2 (4.94–12.46)	0.39
HPV-16	14	3.53 (1.71–5.35)	9	3.8 (2.56–5.04)	5	3.1 (0.41–6.09)	0.73
HPV-52	6	1.51 (0.31–2.71)	4	1.69 (0.06–3.34)	2	1.3	1.000
HPV-68	2	0.50	1	0.42	1	0.63	1.000
HPV-33	3	0.76	2	0.84	1	0.63	1.000
HPV-31	2	0.50	2	0.84	0	—	—
HPV-18	2	0.50	1	0.42	1	0.63	1.000
HPV-58	2	0.50	1	0.42	1	0.63	1.000
HPV-35	1	0.25	1	0.42	0	—	—
HPV-59	1	0.25	1	0.42	0	—	—
HPV-53	1	0.25	1	0.42	0	—	—
HPV-51	1	0.25	1	0.42	0	—	—
HPV-56	2	0.50	1	0.42	1	0.63	1.000
HPV-66	1	0.25	1	0.42	0	—	—
HPV-83	1	0.25	0	—	1	0.63	—
Low risk	14	3.53 (1.71–5.35)	11	4.6 (1.93–7.27)	2	1.26 (0.47–2.99)	0.084
HPV-54	3	0.76	2	0.84	1	0.63	1.000
HPV-55	2	0.50	2	0.84	0	—	—
HPV-11	3	0.76	3	1.26	0	—	—
HPV-42	2	0.50	2	0.84	0	—	—
HPV-40	1	0.25	1	0.42	0	—	—
HPV-43	1	0.25	1	0.42	0	—	—
HPV-44	1	0.25	0	—	1	0.63	—
Multiple infection	18	4.54 (2.49–6.59)	13	5.5 (2.6–8.4)	5	3.2 (0.47–5.93)	0.33
Double infection	14	3.53 (1.71–5.35)	10	4.2 (1.65–6.75)	4	2.5 (0.08–4.93)	0.42
High risk	8	2.02 (0.64–3.4)	5	2.1 (0.28–3.92)	3	1.9 (0.81–2.99)	1.000
HPV-16/58	1	0.25	1	0.42	0	—	—
HPV-16/52	1	0.25	1	0.42	0	—	—
HPV-39/68	1	0.25	1	0.42	0	—	—
HPV-52/58	1	0.25	1	0.42	0	—	—
HPV-18/68	1	0.25	1	0.42	0	—	—
HPV-16/33	1	0.25	0	—	1	0.63	—
HPV-18/58	1	0.25	0	—	1	0.63	—
HPV-52/66	1	0.25	0	—	1	0.63	—
Mixed infection	5	1.26 (0.16–2.36)	4	1.7 (0.06–3.34)	1	0.63 (−0.6–1.86)	0.65
HPV-56/43	1	0.25	0	—	1	0.63	—
HPV-58/6	1	0.25	1	0.42	0	—	—
HPV-52/6	1	0.25	1	0.42	0	—	—
HPV-52/43	1	0.25	1	0.42	0	—	—
HPV-51/54	1	0.25	1	0.42	0	—	—
Low risk	1	0.25	1	0.42	0	—	—
HPV-54/55	1	0.25	1	0.42	0	—	—
Triple infection	4	1.01 (0.03–1.99)	3	1.3 (0.014–2.74)	1	0.63 (−0.6–1.86)	0.65
High risk	2	0.50	1	0.42	1	0.63 (−0.6–1.86)	
HPV-33/35/58	1	0.25	1	0.42	0	—	—
HPV-16/31/52	1	0.25	0	—	1	0.63	—
Mixed infection	2	0.50	2	0.84	0	—	—

**HPV-16/58/44**	1	0.25	1	0.42	0	—	—

^1^Total number of participants.

^2^The HPV prevalence values between the Han and Hani women were compared using the Chi-square test. Bold type indicates statistically significant values.

^3^Percent of positivity (P%) and 95% confidence interval (CI) obtained by using the binomial distribution analysis model.

**Table 2:** Comparison of the age-specific overall, high-risk, single, and multiple HPV infection prevalence in three age groups among the Hani and Han women.

Age	*N* ^6^	Overall	OR 95% CI^5^	*P* value	HR^4^-HPV	OR 95% CI^5^	*P* value	Single	OR 95% CI^5^	*P* value	Multiple	OR 95% CI^5^	*P* value
Hani													
**<35**	65	15	1	0.89	8	1	0.88	11	1	0.64	5	1	0.47
**36–45**	92	19	0.9 (0.4–1.9)		9	0.8 (0.3–2.1)		16	1.1 (0.4–2.4)		3	0.4 (0.1–0.8)	
**>46**	80	16	0.8 (0.4–1.8)		9	0.9 (0.3–2.5)		10	0.7 (0.3–1.8)		5	0.8 (0.2–2.9)	

Han													
**<35**	9	3	1	0.14	2	1	0.22	3	1	**0.07**	1	1	0.42
**36–45**	120	15	0.3 (0.06–1.3)		9	0.3 (0.05–1.6)		10	0.2 (0.03–0.8)		3	0.2 (0.02–2.2)	
**>46**	30	2	0.1 (0.02–1.0)		1	0.1 (0.01–1.5)		2	0.1 (0.01–1.0)		1	0.3 (0.01–4.9)	

^4^HR type was defined as having high-risk (HR) HPV.

^5^Odds ratio (OR) and the 95% confidence interval (CI) were obtained using the logistic regression analysis model. The first category serves as a reference for OR calculation.

^6^Total number of participants.

**Table 3:** Detection of cervical human papillomavirus (HPV) DNA according to potential risk factors in the Hani and Han women.

Variables	Han (*n* ^6^ = 159)				Hani (*n* ^6^ = 237)			*P* value^8^
	Total	Positive	OR^7^ (95% CI)	*P* value	Total	Positive	OR^7^ (95% CI)	
Work				0.95				0.27
Yes	102	13	1		50	6	1	
No	57	7	0.96 (0.23–4.23)		187	44	2.13 (0.55–8.27)	
Age (years)				0.06				**0.05**
<35	9	3	1		65	15	1	
36–45	120	15	0.17 (0.03–1.07)		111	19	0.41 (0.16–1.08)	
>46	30	2	0.06 (0.005–0.64)		61	16	1.09 (0.38–3.17)	
Area				0.91				**0.001**
Rural	36	4	1		168	40	1	
Urban	123	16	1.13 (0.14–9.02)		69	10	0.15 (0.05–0.41)	
Education				0.86				**0.05**
Graduate	61	7	1		23	3	1	
High	25	4	1.43 (0.3–6.77)		21	5	2.21 (0.33–14.68)	
Middle	37	4	0.86 (0.16–4.49)		56	7	0.4 (0.06–2.66)	
Primary	30	5	2.16 (0.3–15.46)		92	24	1.28 (0.19–8.76)	
Illiterate	6	0	—	—	45	11	2.63 (0.36–19.2)	
Married status				0.25				—
Yes	155	19	1		235	50	—	
No	4	1	4.37 (0.36–53.39)		2	—	—	
Sexual partner				0.76				—
Single	137	16	1		234	50	—	
Multiple	24	4	1.25 (0.3–5.17)		3	—	—	
Babies				0.8				**0.02**
No	23	2	1		12	2	1	
Single	87	13	1.98 (0.18–21.97)		73	7	0.93 (0.14–6.09)	
Multiple	49	7	2.55 (0.16–39.41)		152	39	5.35 (0.94–30.47)	
Illness history				0.47				**0.03**
Yes	17	2	1		27	10	1	
No	142	18	2.04 (0.3–13.92)		210	40	3.15 (1.12–8.88)	

^6^Total number of participants.

^7^Odds ratio (OR) and the 95% confidence interval (CI) were obtained using the multiple logistic regression analysis model. The first category serves as a reference for OR calculation.

^8^Bold type indicates statistically significant values.

## Abbreviations

STI: Sexually transmitted infection
HPV: Human papillomavirus
HR-HPV: High-risk human papillomavirus
OR: Odds ratio
CI: Confidence interval.
